# Sex and 30-day survival following out-of-hospital cardiac arrest in Scotland 2011–2020

**DOI:** 10.1186/s12245-024-00731-0

**Published:** 2024-10-07

**Authors:** Laura A. E. Bijman, Sarah H. Wild, Gareth Clegg, Nynke Halbesma

**Affiliations:** 1 Usher Institute, University of Edinburgh, Edinburgh, United Kingdom; 2 Scottish Ambulance Service, Edinburgh, United Kingdom

**Keywords:** Out-of-hospital, Cardiac arrest, Cardiopulmonary resuscitation, Sex, Gender, Survival

## Abstract

**Background:**

Differences in 30-day survival between males and females following out-of-hospital cardiac arrest (OHCA) are well documented. Biological sex does not appear to be responsible for this survival gap independently of potential mediating factors. We investigated the role of potential mediating factors in the association between sex and 30-day survival after OHCA in Scotland.

**Methods:**

A retrospective cohort study of adult non-emergency medical services (EMS)-witnessed OHCA cases was conducted. We included incidents from the whole of Scotland where resuscitation was attempted by the Scottish Ambulance Service (SAS) between April 1, 2011 and March 1, 2020. Logistic regression was used to assess the contribution of age, socioeconomic status, urban–rural location of the incident, initial cardiac rhythm, bystander cardiopulmonary resuscitation (CPR) and location of the arrest (home or away from home).

**Results:**

The cohort consisted of 20,585 OHCA cases (13,130 males and 7,455 females). Median (IQR) age was 69 years (22) for males versus 72 years (23) for females. A higher proportion of males presented with initial shockable rhythm (29.4% versus 12.4%) and received bystander CPR (56.7% versus 53.2%) compared with females. A higher proportion of females experienced OHCA at home (78.8% versus 66.8%). Thirty-day survival after OHCA was higher for males compared with females (8.2% versus 6.2%). Males had higher age-adjusted odds for 30-day survival after OHCA than females (OR, 1.26; (95% CI), 1.12–1.41). Mediation analyses suggested a role for initial cardiac rhythm and location of the arrest (home or away from home).

**Conclusion:**

Males had higher age-adjusted 30-day survival after OHCA than females. However, after adjusting for confounding/mediating variables, sex was not associated with 30-day survival after OHCA. Our findings suggest that initial cardiac rhythm and location of the arrest are potential mediators of higher 30-day OHCA survival in males than females. Improving proportions of females who present with initial shockable rhythm may reduce sex differences in survival after OHCA.

## Introduction

Out-of-hospital cardiac arrest (OHCA) remains a serious health issue worldwide [1]. The global incidence of OHCA ranges anywhere from around 30 to 91.7 per 100,000 population per year [2, 3]. It is well documented that males are more likely to experience OHCA than females [4–6]. For example, a Dutch study using the Amsterdam Resuscitation Studies (ARREST) dataset, reported higher age-standardised incidence rates of OHCA among males versus females (87.3 versus 30.9 per 100,000 person-years) [4] and a population-based cohort dataset from Australia, reported similar findings, with age-standardised incidence for females being almost half of that of males [5]. This sex difference might be partially explained by the higher risk of cardiovascular disease (CVD) in males, this being one of the main causes of OHCA [6, 7]. Differences in risk factors for developing CVD between males and females such as behavioural risk factors, smoking, diabetes, higher levels of cholesterol and blood pressure are also well-established [8, 9].

In Europe, it is estimated that around 8% of patients with OHCA survive to hospital discharge, varying from 0 to 18% between countries [10]. Evidence on differences in survival related to sex is equivocal. Studies report crude 30-day survival rates after OHCA with survival up to twice as high in males than females [11–14]. However some studies have reported that sex differences in survival after OHCA no longer persist after adjustment for factors such as bystander cardiopulmonary resuscitation (bystander CPR, used as a synonym of lay rescuer CPR in this manuscript), initial cardiac rhythm and emergency medical service (EMS) arrival times [12, 15]. This suggests that differences in OHCA survival may not be explained by biological sex and that other arrest characteristics such as bystander CPR, initial cardiac rhythm, age, socioeconomic status or EMS arrival times contribute to the apparent difference in OHCA survival.

In examining OHCA data from Scotland we sought not only to assess whether there was any difference in survival between the sexes but also to see if the data suggested possible causes for any differences found. Variables that distort an association can be classified as either mediating variables or confounding variables. Mediating variables lie on the causal pathway between the dependent and the independent variable [16]. Confounders are associated with the independent and dependent variable separately and do not lie on the causal pathway [17]. Mediating variables that might lie on the causal pathway between sex and survival after OHCA have been proposed [5, 11], but so far the role of individual characteristics has not been examined in detail. This novel approach is important as increasing understanding of factors that may mediate differences in survival between males and females might enable targeted strategies to improve resource allocation. Therefore, the specific aim of this paper was to investigate the impact of potential mediating factors in the sex difference in survival after OHCA, using a large population-based cohort.

## Methods

### Study design and data source

**Fig. 1:**
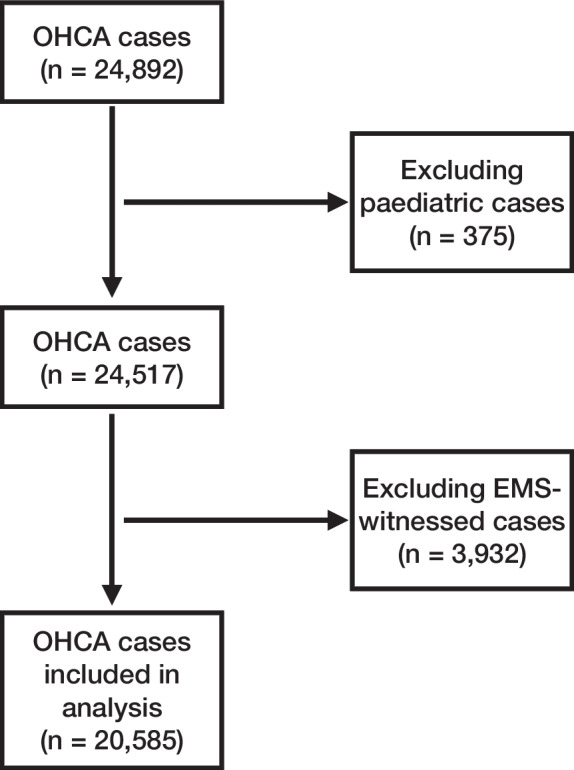
Consort diagram showing the in- and exclusion process of cases

This is a population-based cohort study of non-traumatic, adult OHCA cases where the Scottish Ambulance Service (SAS) attempted resuscitation between April 1, 2011, and March 1, 2020. We decided a priori to exclude cases occurring after March 1, 2020 due to uncertainty of how the COVID-19 pandemic affected the EMS response to OHCA. Paediatric and EMS-witnessed out-of-hospital cardiac arrests were not included in our analysis because they are significantly different from other OHCAs. EMS-witnessed arrests are likely to receive very early advanced life support and are not affected by other elements in the chain of survival e.g. early bystander recognition and call for help or bystander CPR [18]. EMS-witnessed arrests therefore have a much higher likelihood of survival than other OHCAs [18]. Paediatric OHCAs are uncommon and are much more likely to be due to underlying causes associated with poor outcomes [19]. Figure 1 shows the inclusion process via a consort diagram.

**Fig. 2:**
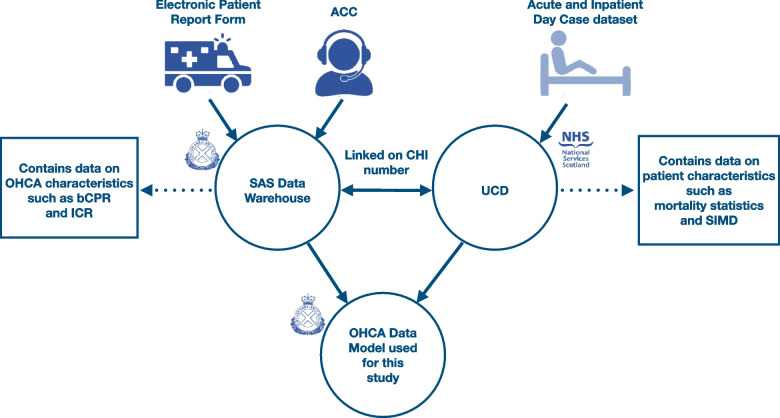
Data collection and linking process to create a cohort containing OHCA cases where resuscitation was attempted by EMS during 2011–2020 in Scotland

Data were collated from various sources. The Utstein OHCA Template guided our data collection process [20]. Data from ambulance service electronic patient report forms and the ambulance control centre was stored in the SAS Data Warehouse. The SAS Data Warehouse contained data on common cardiac arrest characteristics including initial cardiac rhythm, bystander CPR and postcode of the incident (which was used to assign urban–rural classifications [21]). The Unscheduled Care Datamart (UCD) [22] supplied data on common patient characteristics from the Acute and Inpatient Day Case dataset (SMR01) such as the postcode of the individual’s residence (which was used to calculate the Scottish Index of Multiple Deprivation (SIMD) [23] and mortality statistics from the National Records Scotland (NRS) [24]. The National Records Scotland is a database containing information about people living in Scotland, it is a non-ministerial department of the Scottish Government. SAS routinely links the Data Warehouse to the UCD using the patients’ unique Community Health Index (CHI) number [25]. See Fig. 2 for details of the data collation and linking process.

### Variables

The primary outcome was 30-day survival after OHCA. SIMD is an area-based socio-economic deprivation measure used across Scotland [23]. It uses national census data to measure across seven domains: income, employment, education, health, access to services, crime and housing for data zones to create the index [23]. SIMD is based on postcode of the individual’s residence and was described in quintiles with Q1 indicating the most deprived areas and Q5 the least deprived. The postcode for the place where the OHCA took place was classified as either urban or rural using the two-fold Scottish Government Urban Rural Classification [21]. SIMD rankings and Urban Rural Classifications change periodically as census data is updated, and we used the version appropriate for the year of the record for each OHCA incident [23]. Arrival time of the first ambulance was dichotomised at ≤ 8 min and > 8 min according to the Health, Efficiency, Access and Treatment (HEAT) standard on ambulance arrival times used across Scotland [26]. Ambulance arrival times over 30 min were set as missing (*n* = 167). The reason for these lengthy times is usually because the initial coding of the emergency call is not OHCA, but the call is subsequently upgraded (e.g. because the patient arrests while waiting for the ambulance), however the exact cause could not be verified in every case. Location of the arrest (home or away from home) was calculated using the postcode of the incident and the postcode of the patient’s home.

### Statistical methods

We reported age as median with interquartile range (IQR) and the other baseline characteristics of the cohort as frequencies with percentages. We calculated incidence using mid-year population data from the National Records Scotland [27]. We conducted logistic regression analyses to describe the crude and age-adjusted association between sex and 30-day survival after OHCA reported as odds ratio (OR) with 95% confidence interval (CI). We have checked the regression assumptions. To assess the impact of potential mediators, we used logistic regression by creating separate models, each model adjusting for one different potential mediator at a time. We followed ‘the difference-between-coefficients’ approach or causal mediation first introduced by Baron & Kenny [28] as part of Structural Equation Modelling (SEM) [29, 30]. This approach is useful for simple mediation analyses [29, 31]. There can be no unmeasured exposure-outcome confounding, no unmeasured mediator-outcome confounding and no unmeasured exposure-mediator confounding when using this method. We assessed all potential mediators before performing causal mediation analysis. The relative change in OR (%) was reported for each potential mediator when compared with the first model (age-adjusted) [32]. The cut-off point for describing a variable as a mediator was set at a change of more than 10% [33]. We conducted sensitivity analyses by creating subgroups of males and females with initial shockable rhythm, males and females who received bystander CPR and males and females who experienced OHCA at home to assess whether the age-adjusted survival difference found between sexes could be explained by either of these three variables as they are important predictors for survival after OHCA. The statistical software package R version 4.0.3 was used for all analyses [34].

### Ethical approval

This project was given approval by the Scottish Ambulance Service Research & Development Committee (reference number: SASRD-2020-011) and submitted to the Health Research Authority’s decision tool. As this is a non-transferable analysis of routinely collected data, separate ethical review was not required.

## Results

### Study population

Our study population consisted of 20,585 adult OHCA cases where resuscitation was attempted by EMS occurring between April 1st 2011 and March 1st 2020. EMS-witnessed arrests were excluded (*n* = 3,932). The proportion of missing data was low (1.6% of the dataset, *n* = 326). Comparison between included and excluded cases due to missingness did not show any differences in baseline characteristics.

 Table 1 shows the baseline characteristics of the cohort including numbers of missing data. The overall incidence of OHCA in Scotland from 2011–2020 was 42.5 per 100,000 population per year. Incidence of OHCA was higher in males than in females (55.8 versus 29.9 per 100,000 population per year). The proportion with initial shockable rhythm was 29.4% in males versus 17.4% in females. Bystander CPR was received by 56.7% of males versus 53.2% of females. Thirty-day survival after OHCA was 8.2% in males and 6.2% in females. The proportion of OHCA incidents occurring at home was 66.8% in males and 78.8% in females.

**Table 1:** Baseline characteristics of cohort containing EMS attended OHCA cases where resuscitation was attempted by EMS during 2011–2020 in Scotland

	Total (*n* = 20,585)	Male (*n* = 13,130)	Female (*n* = 7,455)	Missing N (%)
Incidence per 100,000 population per year	42.5	55.8	29.9	
Age, median (IQR)	70.0 (22.0)	69.0 (22.0)	72.0 (23.0)	
SIMD, n (%)				321 (2)
Most deprived Q1	5,453 (26.9)	3,314 (25.7)	2,139 (29.1)	
Q2	4,866 (24.0)	3,048 (23.6)	1,818 (24.7)	
Q3	3,902 (19.3)	2,542 (19.7)	1,360 (18.5)	
Q4	3,251 (16.0)	2,139 (16.6)	1,112 (15.1)	
Least deprived Q5	2,792 (13.8)	1,870 (14.5)	922 (12.6)	
Urban/Rural, n (%)				201 (1)
Urban	14,795 (72.6)	9,304 (71.6)	5,491 (74.3)	
Rural	5,589 (27.4)	3,685 (28.4)	1,904 (25.7)	
Initial shockable rhythm, n (%)				
No	15,436 (75.0)	9,275 (70.6)	6,161 (82.6)	
Yes	5,149 (25.0)	3,855 (29.4)	1,294 (17.4)	
Bystander CPR, n (%)				
No	9,181 (44.6)	5,691 (43.3)	3,490 (46.8)	
Yes	11,404 (55.4)	7,439 (56.7)	3,965 (53.2)	
Location of the arrest				267 (1)
Home	14,453 (70.2)	8,653 (66.8)	5,800 (78.8)	
Away from home	5,865 (28.5)	4,302 (33.2)	1,563 (21.2)	
EMS arrival time (mins), n (%)				180 (1)
Median (IQR)	7.6 (5.2)	7.6 (5.3)	7.6 (5.2)	
≤ 8	11,049 (54.1)	7,070 (54.3)	3,979 (53.8)	
> 8–29	9,356 (45.9)	5,944 (45.7)	3,412 (46.2)	
Survival to 30 days, n (%)				
No	19,045 (92.5)	12,051 (91.8)	6,994 (93.8)	
Yes	1,540 (7.5)	1,079 (8.2)	461 (6.2)	

*Abbreviations*: *CPR* cardiopulmonary resuscitation, *EMS* emergency medical service, *IQR* interquartile range, *SD* standard deviation, *SIMD* Scottish index of multiple deprivation, *Q* quintile

Crude OR for 30-day survival after OHCA was 1.36; (95% CI) 1.21–1.52 for males compared to females. Age-adjusted OR for 30-day survival after OHCA was 1.26; (95% CI) 1.12–1.41 for males compared to females.

**Fig. 3:**
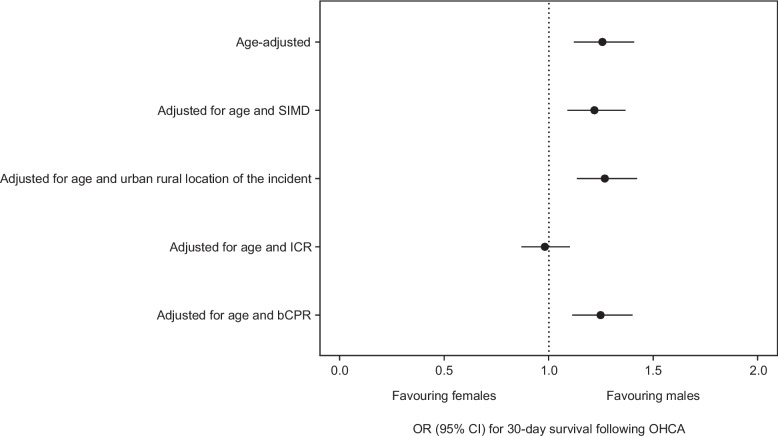
Forest plot showing odds ratios for 30-day survival following OHCA where resuscitation was attempted by EMS for males compared to females in Scotland 2011–2020 estimated from different logistic regression models

Following inclusion of all potential confounders and mediators, the OR for 30-day survival for males compared to females was 0.83; (95% CI), 0.74–0.94. There was evidence of mediation by initial cardiac rhythm (OR 0.98; (95% CI), 0.87–1.10) and location of the arrest (OR 1.08; (95% CI), 0.96–1.22) (Table 2, Fig. 3) shown by the attenuation of the OR for 30-day survival for males compared to females.

**Table 2:** Odds ratios for 30-day survival following OHCA where resuscitation was attempted by EMS for males compared to females in Scotland 2011–2020 estimated from different logistic regression models

	OR (95% CI)	% change in OR^a^
Unadjusted	1.36 (1.21–1.52)	N/A
Age-adjusted	1.26 (1.12–1.41)	N/A
Adjusted for age + SIMD	1.22 (1.09–1.37**)**	3.2
Adjusted for age + urban/rural location of the incident	1.27 (1.13–1.42)	0.8
Adjusted for age + initial cardiac rhythm	0.98 (0.87–1.10)	22.2
Adjusted for age + bystander CPR	1.25 (1.11–1.40)	0.8
Adjusted for age + location of the arrest	1.08 (0.96–1.22)	14.3
Adjusted for age, SIMD, urban/rural location of the incident, initial cardiac rhythm, bystander CPR and location of the arrest	0.83 (0.74–0.94)	N/A

*Abbreviations*: *CPR* cardiopulmonary resuscitation, *CI* confidence interval, *N/A* not applicable, *OR* odds ratio, *SIMD* Scottish index of multiple deprivation

^a^All logistic regression models were compared with the age-adjusted logistic regression model to calculate % change in OR

### Post-hoc analyses

Results of post-hoc analyses (all age-adjusted) in separate strata of receipt/non-receipt of bystander CPR, presence/absence of initial shockable rhythm and location of arrest at home/away from home can be found in Table 3. In both subgroups of initial shockable rhythm (*n* = 5,149) and initial non-shockable rhythm (*n* = 15,436), we did not find any association between sex and 30-day survival after OHCA. In the subgroup of patients who did not receive bystander CPR (*n* = 9,181), we did not find any association between sex and 30-day survival after OHCA. In the subgroup of patients who received bystander CPR (*n* = 11,404), 30-day survival was markedly higher in males than females. However, when adjusted for initial shockable rhythm, the association disappears (OR 1.09; (95% CI) 0.94–1.28). In the subgroup of patients who experienced OHCA at home (*n* = 14,453), we did not find any association between sex and 30-day survival after OHCA. In the subgroup of patients who experienced OHCA away from home (*n* = 5,865), 30-day survival was markedly higher in males than in females.

**Table 3:** Post-hoc analyses for 30-day survival following OHCA where resuscitation was attempted by EMS for males compared to females in Scotland 2011–2020 estimated from different logistic regression models in different subgroups

	N	OR (95% CI) (age-adjusted)
Subgroup initial non-shockable rhythm	15,436	1.05 (0.88–1.25)
Subgroup initial shockable rhythm	5,149	0.92 (0.78–1.09)
Subgroup no bystander CPR	9,181	0.96 (0.80–1.16)
Subgroup bystander CPR	11,404	1.44 (1.25–1.67)
Subgroup home	14,453	0.95 (0.81–1.12)
Subgroup away from home	5,865	1.26 (1.06–1.50)

*Abbreviations*: *CPR* cardiopulmonary resuscitation, *CI* confidence interval, *OR* odds ratio

## Discussion

The overall incidence of OHCA in Scotland from 2011–2020 was 42.5 per 100,000 population per year. Incidence of OHCA was higher in males than in females (55.8 versus 29.9 per 100,000 population per year), consistent with sex differences reported previously [4, 5]. In countries where CVD is a common cause of death, the incidence of OHCA is generally higher in men [4, 5]. Our analyses indicated that in a large population-based cohort of OHCA cases, males had higher proportions of OHCAs presenting with initial shockable rhythm (29.4% versus 17.4%), higher proportions receiving bystander CPR (56.7% versus 53.2%) and higher 30-day survival after OHCA compared with females (8.2% versus 6.2%). Females were more likely to experience OHCA at home (78.8% versus 66.8%). These patterns are consistent with reports from other studies [11, 35, 36]. Age-adjusted 30-day survival after OHCA was higher among males than females. This is in line with a recent study by Chen et al. (2024) [37]. However, after adding all potential confounding and mediating variables into a logistic regression model, there was no longer a direct association between biological sex and survival after OHCA. This was consistent with the findings in two recently published systematic reviews [15, 38].

We found that initial cardiac rhythm and location of the arrest appeared to mediate the association between sex and 30-day survival following OHCA with bystander CPR possibly contributing to the sex difference to a smaller extent. Post-hoc analyses supported this finding as the association between sex and 30-day survival was absent in the subgroup who had initial shockable rhythm and the subgroup of patients experiencing OHCA at home. Higher 30-day survival following OHCA was only observed in males compared to females in the subgroup that received bystander CPR suggesting bystander CPR may mediate the association between sex and 30-day survival after OHCA. However, when adjusted for initial shockable rhythm the association disappears. This indicates that patients who receive bystander CPR are more likely to present with initial shockable rhythm. Various studies confirm this [39]. Other factors such as urban/rural location of the incident and socio-economic status did not appear to confound or mediate the association between sex and survival found in the age-adjusted analysis. Two recent studies seem to support these findings. Wittwer et al. (2022) [5] and Rob et al. (2022) [11] reported that sex differences in outcome of EMS-treated OHCA appear to be driven by differences in initial shockable rhythm rather than treatment delays or other disparities between males and females. However, both of these studies did not specifically investigate mediation and made suggestions based on differences in crude proportions of variables. Wittwer et al. (2022) used a small cohort containing 780 EMS treated OHCA cases in Australia [5]. They did not report a statistically significant difference in proportions of bystander CPR between males and females (59% in males versus 54% in females), however, they reported an almost 50% difference in proportions of initial shockable rhythm between males and females (32% in males versus 19% in females) [5]. This is similar to our findings. In a small cohort of 932 OHCA patients admitted to hospital, Rob et al. (2022) [11] reported that proportions of bystander CPR were 86% in males versus 82% in females whereas initial shockable rhythm was 65% in males versus 47% in females [11]. The higher proportions that had received bystander CPR in this cohort possibly reflects the selection of a subgroup that was admitted to hospital. Life expectancy is higher in females compared with males, as people grow old they are less likely to get out of the house and e.g. reside in a nursing home [40]. This could explain why a higher proportion of females experience OHCA at home compared with males. Experiencing OHCA at home likely leads to unfavourable OHCA characteristics such as having an unwitnessed arrest, not receiving bystander CPR and no bystander defibrillator use [41].

Furthermore, aetiology of the OHCA is an important predictor of initial shockable rhythm [42, 43]. Males have higher incidence of myocardial infarction than females, whereas females are more likely to experience OHCA due to e.g. pulmonary embolism [44, 45]. Different aetiologies of OHCA may lead to differences in initial cardiac rhythm, with males possibly therefore more likely to present with initial shockable rhythm [6].

Our study is one of the first to examine mediators when describing the association between biological sex and 30-day survival after OHCA and to highlight the important difference between mediators and confounders when investigating outcomes among patients receiving care from emergency medical services. The mediation analysis performed in this paper used the simple Baron & Kenny (1986) [28] method for investigating causal mediation and is likely to have met the required assumption that there are no major unmeasured confounders because biological sex cannot generally be changed or influenced after birth (unless undergoing specific surgery or being intersex) [46, 47]. To our knowledge, the extent to which the difference between male and female survival after OHCA is explained by differences in initial shockable rhythm has not previously been reported.

### Limitations

There are several limitations to our study. First, excluding OHCA cases occurring during the COVID-19 pandemic (after March 1, 2020) made the dataset less contemporary and may not reflect patterns during the pandemic. Second, there could have been some residual confounding, as data on lifestyle factors such as obesity, smoking and alcohol consumption was not available. Third, SIMD is an area-based variable therefore it does not always accurately reflect individual socioeconomic status. Another limitation is that human error might have occurred as pre-hospital variables such as whether or not bystander CPR was present were manually entered by EMS personnel and difficult to verify subsequently. Data on whether or not arrests happened in a nursing home, whether or not arrests were witnessed, co-morbidities, cause of the arrest and bystander AED use were not available and we were unable to include these variables in our analysis. These variables may also account for some of the difference in 30-day survival after OHCA between males and females. In particular aetiology of cardiac arrest is an important omission as literature suggests it differs between males and females and influences whether or not anyone presents with an initial shockable rhythm after OHCA. Another limitation is that we were not able to investigate sex differences in in-hospital treatment. Finally, there are more advanced mediation methods available than the one used here, but results are more difficult to interpret than those from simple approaches [32, 48]. As Scotland is a country with very rural regions (reflected in response times for example), caution should be taken into account when generalising the results of this study to other countries.

### Clinical implications

Initial shockable rhythm may be influenced by additional factors beyond aetiology of the OHCA [49, 50] such as early bystander CPR, rapid deployment of public access defibrillators (PADs) and rapid EMS response times [51]. Delays in initiating treatment such as bystander CPR and deploying a PAD may result in a shockable rhythm becoming a non-shockable rhythm [51]. Timely bystander CPR and efficient PAD use could increase the percentage of patients presenting with initial shockable rhythm [51].

## Conclusions

Higher proportions of initial shockable rhythm and bystander CPR among males, as well as greater proportions of females experiencing OHCA at home, contribute to the higher 30-day survival rates in males compared to females. Early bystander CPR and rapid deployment of automated external defibrillators may increase the proportion of females presenting with initial shockable rhythm, lead to successful defibrillation and subsequently narrow the survival gap between males and females following OHCA.

## Data Availability

Due to the nature of the research and due to ethics, supporting data is not readily available. However upon reasonable request data can be obtained from the corresponding author.

## Abbreviations

ARREST: Amsterdam resuscitation studies
CHI: Community health index
CI: Confidence interval
CPR: Cardiopulmonary resuscitation
CVD: Cardiovascular disease
EMS: Emergency medical services
HEAT: Health, efficiency, access and treatment
IQR: Interquartile range
NRS: National records Scotland
OHCA: Out-of-hospital cardiac arrest
OR: Odds ratio
PAD: Public access defibrillator
SAS: Scottish ambulance service
SIMD: Scottish index of multiple deprivation
UCD: Unscheduled data mart
